# The effect of intra-vaginal oxytocin on sexual function in breastfeeding mothers: a randomized triple-blind placebo-controlled trial

**DOI:** 10.1186/s12884-022-04384-w

**Published:** 2022-01-22

**Authors:** Arezu Mesbahi, Sakineh Mohammad-Alizadeh-Charandabi, Zahra Ghorbani, Mojgan Mirghafourvand

**Affiliations:** 1 Department of midwifery, Student Research Committee, Faculty of Nursing and Midwifery, Tabriz University of medical sciences, Tabriz, Iran; 2 Midwifery Department, Faculty of Nursing and Midwifery, Tabriz University of Medical Sciences, Tabriz, Iran; 3 Faculty of Nursing and Midwifery, Tehran University of Medical Sciences, Tabriz, Iran; 4 Social Determinants of Health Research Center, Faculty of Nursing and Midwifery, Tabriz University of Medical Sciences, Tabriz, Iran

**Keywords:** Intravaginal Oxytocin, Sexual function, Sexual dysfunction, Breastfeeding mothers, Postpartum

## Abstract

**Background:**

Considering the importance of sexual function, high prevalence of sexual dysfunction (especially dyspareunia caused by atrophic vaginitis) in breastfeeding women, and lack of effective interventions, the present research aimed to determine the effect of oxytocin (OXT) vaginal gel on sexual function (primary outcome), sexual satisfaction, and depression (secondary outcomes) in the breastfeeding women.

**Methods:**

This randomized triple-blind controlled trial was conducted on 64 breastfeeding women who referred to health centers in the city of Tabriz, Iran, in 2020-21. Participants were equally assigned to intervention/control groups using block randomization. 200 IU of OXT vaginal gel was given to the participants in the intervention group daily for eight week and the same protocol was carried out for the control group with placebo. Standard questionnaires of Female Sexual Function Index (FSFI), Edinburgh Postpartum Depression Scale (EPDS) and Sexual satisfaction scale for women (SSSW) were completed at baseline and 8 weeks after intervention. ANCOVA test was used to compare post-intervention mean score of the groups, adjusted for the baseline values.

**Results:**

After intervention, there was no statistically significant difference between groups in terms of mean total score of FSFI (Adjusted Mean Difference (AMD): 1.14; 95% Confidence Interval (95% CI): -1.28 to 9.16; P= 0.349) and sexual satisfaction (AMD: 5.01; 95% CI: -0.53 to 10.56; P= 0.075). However, there was statistically significant difference between the groups in terms of mean scores of sexual contentment (AMD: 1.56; 95% CI: 0.29 to 2.83; P = 0.017) and depression (AMD: -1.90; 95% CI: -1.27 to -2.54; P < 0.001). One participant in the OXT group and one participant in the placebo group reported mild uterine contraction and one person in the placebo group reported vaginal burning sensations.

**Conclusions:**

No evidence was found for the effects of OXT gel in the improvement of FSFI, even though, OXT significantly improved sexual satisfaction in the domain of contentment, and improved the symptoms of depression in comparison to the placebo group. However, a definite conclusion requires more research in this regard.

**Trial registration:**

the Iranian Registry of Clinical Trials (IRCT), code: IRCT20120718010324N55, Date of registration: 27/05/2020, URL: https://en.irct.ir/user/trial/44986/view.

**Supplementary Information:**

The online version contains supplementary material available at 10.1186/s12884-022-04384-w.

## Background

Pregnancy and childbirth cause hormonal and physical changes in the person and exert considerable effects on the health of the mothers and the quality of their lives [1]. Sexual function undergoes changes during the disparate periods of a woman’s life and one of these periods that is less considered is the postpartum period [2]. The changes during the postpartum period include pain during coitus, reduction or lack of sexual desire, and failure to achieve orgasm [3]. The studies indicated that 91% of women suffer from sexual problems during the postpartum period, especially loss of sexual desire, dyspareunia, and atrophic vaginitis [4, 5].

Sexual satisfaction is one of the physiological needs that leads to human health. Lack of sexual satisfaction, and physical and psychological pressures caused by it can result in disorders and reduce a person’s capabilities as well as creativity [7]. Sexual dysfunction is a chain of sexual-psychological disorders [6]. The results of the review of the literature indicated that there is a bilateral relationship between female sexual dysfunction (FSD) and depression [8–10]. Both sexual dysfunction and depression are more prevalent in women, during the postpartum period [11, 12]. The results of Natsal-3 cohort study showed that depression is one of the most prevalent common diseases with FSD [13]. As a result, simultaneous screening in people with FSD and women who gave birth recently is considerably important [3].

A variety of methods were employed in order to prevent and treat FSD. Hormone replacement therapy (HRT) is one of the most common methods for improving the sexual function. However, these drugs (especially estradiol) cannot be used by breastfeeding mothers since they have a considerable impact on the quality and quantity of the breast milk. Treatment with topical lubricant gels is one of the methods used during breastfeeding with no enough effect [7]. One of the hormones recently studied by researchers to treat sexual disorder is the oxytocin (OXT) hormone. Besides fewer side effects, this method can lead to more benefits than the common systematic and topical treatments [14–18].

OXT is a nine-amino-acid peptide hormone produced in the supraoptic and paraventricular nucleus of the hypothalamus and released into blood flow through the posterior pituitary [18]. The most known performance of OXT is during the start and progression of the process of childbirth and facilitation of milk secretion after childbirth [16]. OXT has a crucial role in regulating the mood, forming social interactions, mother-child attachment, and creating a sense of trust and intimacy in the interpersonal and marital relationship. OXT operates centrally in the central nervous system and environmentally in various tissues such as the ovary, testis, gastrointestinal system, and cardiovascular system through OXT receptors [19–22].

Several studies argued that disruption in the hormones secreted from the hypothalamic–pituitary–adrenal (HPA) axis can cause sexual dysfunction. Some studies have shown positive effect of intranasal OXT on the intensity of orgasm, arousal, and sexual satisfaction in women and men with sexual dysfunction [23–26]. One of the key mechanisms of OXT is stimulating the growth of natural cells in the body, which operates as a growth factor similar to growth factors of nerves and insulin. Furthermore, in the laboratory and animal tests, OXT restored the ulcers and damages to environmental nerves and recovered the skin and epithelium of the vagina through anti-inflammatory processes and secreting inflammatory cytokines. OXT accelerates the scar healing process of various cells through mechanisms such as increasing the tissue perfusion, increasing the secretion of the disparate growth factors, and stimulation of mitosis. Effective factors on reducing or improving the symptoms of atrophic vaginitis might follow a similar path [18, 27].

Taking into account the already stated mechanism of action of this hormone, psychologists and sex therapists recently become interested in this subject. Therefore, prescribing exogenous OXT may reduce and improve sexual disorders, especially sexual dysfunction caused by atrophic vaginitis in breastfeeding women. The present research aimed to determine the effect of OXT vaginal gel on sexual function (primary outcome), sexual satisfaction, and depression (secondary outcomes) in the breastfeeding mothers.

## Methods

### Study design and participants

This is a randomized, placebo-controlled, triple-blind trial. The trial conducted on breastfeeding women covered by the health centers in the city of Tabriz, Iran, in 2020-21. The participants, outcome assessors, and data analysts were not aware of the interventions received by each participant.

Participants were married healthy breastfeeding women (with no records of childbirth complications or illness), with a constant and healthy sexual partner, were sexually active, and 6 weeks to 6 months had passed from their childbirth. Women who obtained a score of less than 28 in the Female Sexual Function Index (FSFI) and a score of less than 13 in the Edinburgh Postpartum Depression Scale (EPDS) were included in the study. Use of highly effective contraceptive method was another inclusion criterion. The exclusion criteria were being pregnant, having postpartum depression, consumption of antidepressants and other nervous system drugs, hormonal treatment due to female sexual dysfunction, pelvic radiotherapy and chemotherapy, or any uncontrolled and chronic disease such as diabetes.

### Sample size

The sample size was calculated in accordance with the sexual function variable and using G-Power software. According to the study by Sehhatie et al., [28] regarding the sexual function and by considering M_1_=16.4 (pre-intervention mean) and M_2_=19.68 (assuming a 20% increase due to intervention), SD_1_=SD_2_=4.3, α = 0.05, Power*=*90%, sample size was calculated to be equal to 31 participants in each group. The total final sample size amounted to 34, taking into account 10% attrition.

### Sampling

After approval of the Ethics Committee of Tabriz University of Medical Sciences (IR.TBZMED.REC.1396.715), and registration of the study in the Iranian Registry of Clinical Trials (IRCT20120718010324N32), the researcher attended the health centers in the city of Tabriz-Iran for sampling. There are 80 health centers in Tabriz city. Participants were selected from the ten crowded centers with different socio-economic classes. Information of all women, either pregnant or who gave birth, was available in the household health information system. The principal investigator (AM) extracted phone number of potentially eligible women from the system, contacted and explained them the objectives of the study, and invited them to attend the health center. Women who were willing to participate were included in the study after assessing the inclusion and exclusion criteria and obtaining the written informed consent. Then, the scales were completed by all participants. Furthermore, anthropometric indices were calculated including weight and height (to measure the body mass index (BMI)), waist circumference, and blood pressure.

### Randomization and intervention

The participants were randomly assigned into two groups (recipient of OXT or placebo vaginal gel) via the www.random.org website and using block randomization with block sizes of 8 and 10. In order to conceal allocation, the drug/placebo was packaged in identical consecutively numbered packages. The allocation generation and preparation of the packages were carried out by a person not involved in the processes of data collection and analysis.

Raw materials of OXT were supplied from the Caspian Tamin Pharmaceutical Company. The gel and the placebo were prepared in the Faculty of Pharmacy of Tabriz University of Medical Science. Placebo was a water-based gel with neutral pH that was contained carbomer 934, Methyl and propyl paraben as a preservative (prevent from growth of microorganisms). OXT gel was exactly the same as placebo, except that it contains 200 IU of the active ingredient OXT powder. Each IU of OXT is equivalent to 1.67 µg, so 200 IU of OXT is equal to 344 µg. The placebo gel and tubes were exactly the same color, odor and size as OXT gel and tubes. In the present research, 200 IU of OXT vaginal gel was given to the women in the intervention group once a day for eight weeks and the same protocol was carried out for the control group with placebo drug. Drug/placebo gel was used by the participants using applicators at any hour of the day and with the maximum of half an hour prior to sexual intercourse. Four OXT or placebos gel tubes were provided for each participant, and an OXT or placebo gel tube were delivered to the participants once every two weeks in the health centers. Therefore, the investigator followed-up the participants once every two weeks to remind and to emphasize regular consumption of the drugs.

### Tools

The data were collected using the sociodemographic and obstetrics information questionnaire, FSFI, Sexual satisfaction scale for women (SSSW), EPDS, and a checklist of the side effects. The Persian version of the EPDS, FSFI and SSSW have been approved in Iran after obtaining permit from the copyright holders of the English version of the scales. The used scales are available as Appendix.

### FSFI

FSFI is a standard and international self-report tool used for the assessment of the sexual function of women, which was first designed by Rosen [29]. This questionnaire contains 19 items that measure women’s sexual function in six domains. Each domain includes the following weight coefficient and a number of items. Sexual desire (2 items*0.6), arousal (4 items*0.4), lubrication (4 items*0.4), orgasm (3 items*0.3), satisfaction (3 items*0.3), and pain (3 items*0.3). The total score of FSFI was obtained from the sum of the scores at each domain and considering their specific coefficients. The total sexual function score ranged between 2 and 36. FSFI<28 was considered as sexual dysfunction. In this index, the highest score shows the desirable sexual function. The validity and reliability of FSFI were confirmed by Fakhri et al. and Mohammadi et al. in Iran [30, 31].

### SSSW

In the present research, the SSSW was used to measure women’s sexual satisfaction, which included 30 questions in five domains: contentment (questions 1 to 6), communication (questions 7 to 12), compatibility (questions 13 to 18), relational concern (questions 19 to 24), and personal concern (questions 25 to 30). In this questionnaire, the 5 points Likert scale was used, i.e. absolutely agree 1 point, agree 2 points, I don’t know 3 points, disagree 4 points, absolutely disagree 5 points. Inverted scoring was used for questions 1, 4, 5, 6, 9, 10, 11, and 12. The score of each domain were obtained using sum score of related questions; with the higher score, the more sexual satisfaction. The validity and reliability of this tool were confirmed by Roshan Chesli et al. [32].

### EPDS

EPDS was employed for the assessment of depression. It was designed by Cox et al. [33] and is used to measure pregnancy and postpartum depression. This tool includes 10 questions with four options scored 0-3. In some questions, the order of the options was from less to high severe (items 1, 2, and 4) and some were ordered from high to low (items 10, 9, 8, 7, 6, 5, and 3). The total score which is varied from zero to 30, is obtained from summing up the question scores. Those with score of higher than 12 suffer from depression. The validity and reliability of this scale were approved by Montazeri et al.  [34].

### Checklist of side effects

In the present research, the side effects were extracted through searching from scientific data bases and literature review and were listed in a checklist which included nausea, vomiting, headache, uterine contractions, vaginal itching and burning sensations. Any reported side effects by participants were also recorded.

### Medication recording checklist

To assess compliance with the medication, the participants were requested to record their intake of the gel in a medication recording checklist. The checklist was provided to participants in the first visit. Also for assessing adherence to treatments, telephone calls were made weekly to emphasis the regular use of the medications and assess the side effects and potential withdrawals.

### Data analysis

The data were analyzed using SPSS software version 24. Kolmogorov-Smirnov test was used to determine the normality of the quantitative data. All data had normal distribution. After the intervention, ANCOVA test adjusted for the baseline scores was used to compare the mean scores of sexual function, sexual satisfaction and depression between groups. Mann Whitney U test was used to compare the change in depression score (EPDS at 8 weeks - EPDS at baseline) between the two groups as a sensitivity analysis to confirm the results. Subgroup analysis was done based on the delivery type by using ANCOVA test. The Mann Whitney U test and subgroup analyses were post-hoc, following reviewers’ suggestions. The level of significance was considered less than 0.05.

## Results

### Baseline characteristics

**Fig. 1:**
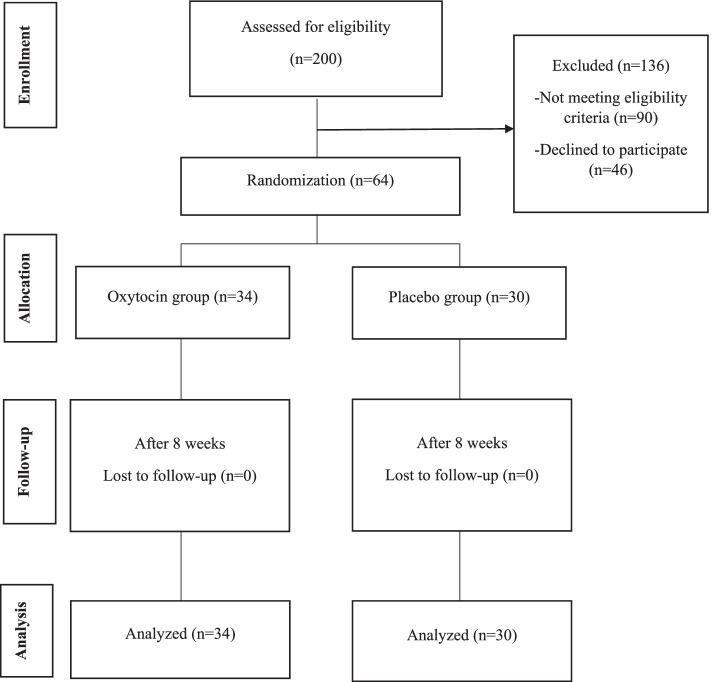
Flowchart of the study

All 200 breastfeeding women selected from the health centers’ electronic system underwent screening in terms of eligibility criteria in the first assessment. Among them, 44 women were excluded from the study due to medical problems and childbirth complications, consumption of drugs affecting sexual function, and problems pertinent to the child, and 46 people were not interested in participating in the study. 64 out of 100 participants who fulfilled the FSFI questionnaire were identified as having sexual dysfunction (FSFI<28) and included in the study. The rest of questionnaires were completed after obtaining informed written consent. The participants were randomly assigned into two groups, i.e. OXT vaginal gel group (n = 34) and the placebo group (n = 30). There was no loss to follow-up and all participants in both groups continued the intervention up to eight weeks. After completion of the study, all questionnaires were filled in by the participants (Fig. 1).

Table 1 shows the socio-demographic and obstetrics characteristics of the participants by their groups. There was no statistically significant difference between the two groups except of age (P > 0.05). The variable of age was adjusted in the ANCOVA models.

**Table 1:** Socio-demographic and obstetrics characteristic of the participants in the study groups

Variables	Oxytocin (n = 34)	Placebo (n = 30)	
	Number (Percent)	Number (Percent)	
**Age** (year) ^a^	31.2 (5.1)	27.8 (5.9)	
**Level of education**			
Illiterate	1 (2.9)	2 (6.7)	
Elementary	6 (17.6)	1 (3.3)	
Secondary	5 (14.7)	10 (33.3)	
High school	3 (8.8)	8 (26.7)	
Diploma	9 (26.5)	5 (16.7)	
University	10 (29.4)	4 (13.3)	
**Spouse’s Education**			
Illiterate			
Elementary	6 (1.6)	6 (20.0)	
Secondary	7 (20.6)	7 (23.3)	
High school	5 (14.7)	2 (6.7)	
Diploma	6 (17.6)	10 (33.3)	
University	10 (29.4)	5 (16.7)	
**Job**			
Housekeeper	27 (79.4)	28 (93.3)	
Employed indoors	1 (2.9)	1 (3.3)	
Employed outdoors	6 (17.6)	1 (3.3)	
**Spouse’s Job**			
Unemployed	0 (0)	1 (3.3)	
Worker	4 (11.8)	10 (33.3)	
Retired	1 (2.9)	0 (0)	
Self employed	20 (58.8)	15 (50)	
Private sector employee	2 (5.9)	0 (0)	
Public sector employee	7 (20.6)	4 (13.3)	
**Parity**			
1	11 (32.4)	9 (30.1)	
2	12 (35.3)	18 (60)	
3	8 (23.5)	3 (10)	
4	2 (5.9)	0 (0)	
5	1 (2.9)	0 (0)	
**Delivery type**			
Vaginal with episiotomy	10 (29.4)	14 (46.7)	
Vaginal without episiotomy	0 (0)	3 (10.0)	
Elective cesarean section	16 (47.1)	9 (30.0)	
Emergency cesarean section	8 (23.5)	4 (13.3)	
**Abortion**			
Yes	10 (29.4)	13 (43.3)	
No	24 (70.6)	17 (56.7)	
**Family Members number**			
1-3	21 (61.8)	17 (56.7)	
4-6	11 (32.4)	10 (3.33)	
>6	2 (5.9)	3 (10)	
**Sufficiency of monthly income**			
Inadequate	7 (20.6)	7 (23.3)	
Relatively adequate	25 (73.5)	21 (70)	
More than adequate	2 (5.9)	2 (6.7)	
**Life satisfaction**			
Satisfied	28 (482)	29 (96.7)	
No idea	4 (11.8)	14 (3.3)	
Dissatisfied	2 (5.9)	0 (0)	
**Live with spouse’s family**			
Yes	6 (17.6)	11 (36.7)	
No	28 (82.4)	19 (63.3)	
**Spouse’s smoking**			
Yes	11 (32.4)	11 (36.7)	
No	23 (67.67)	19 (63.3)	
**Infant other nutrition**			
Yes	20 (58.8)	11 (36.7)	
No	14 (41.2)	19 (63.3)	
**Infant age** (Month)	4.18 (1.88)	3.87 (1.72)	
Waist circumference^a^ (cm)	77.18 (13.06)	75.50 (11.69)	
Hip circumference^a^ (cm)	93.35 (10.92)	92.03 (8.22)	
Blood pressure (mmHg)			
Systole^a^	111.32 (9.32)	111.00 (11.73)	
Diastole^a^	68.23 (11.21)	70.00 (11.45)	
Body mass index^a^ (kg/m^2^)	25.65 (2.75)	25.28 (3.94)	

^a^ The numbers were reported as mean (standard deviation)

### Sexual function

The mean (SD) total score of FSFI in the OXT group was 19.48 (4.68) before the intervention and 27.50 (5.12) after the intervention, and in the placebo group, it was 22.28 (4.15) before the intervention and 28.14 (4.77) after the intervention. Based on the ANCOVA test with adjusting the baseline value and age, there was no statistically significant difference between groups in terms of the total score of sexual function (AMD: 1.14; 95% CI: -1.28 to 9.16; P= 0.349). Table 2 shows the mean total score of sexual function and all of its domains by the two groups, before and after the intervention.

**Table 2:** The mean FSFI total score and its dimensions in study groups

Variable (score range)	Oxytocin n=34	Placebo n= 30	AMD (95% CI)^b^	P-value
	Mean (SD)^a^	Mean (SD)^a^		
**Desire (1.2-6)**				
Baseline	2.91 (0.96)	3.42 (0.74)		
8 weeks	4.38 (1.00)	4.34 (1.13)	0.32 (0.86 to -0.23)	0.253
**Arousal (0-6)**				
Baseline	1.04 (2.98)	3.49 (0.74)		
8 weeks	4.41 (1.09)	4.46 (1.04)	0.32 (0.81 to -0.18)	0.208
**Lubrication (0-6)**				
Baseline	3.34 (0.85)	3.60 (0.8)		
8 weeks	4.53 (0.87)	4.65 (0.81)	0.06 (0.47 to -0.36)	0.791
**Orgasm (0-6)**				
Baseline	3.21 (1.00)	2.76 (1.1)		
8 weeks	4.46 (1.00)	4.53 (1.03)	0.34 (0.77 to 0.08)	0.114
**Satisfaction (0.8-6)**				
Baseline	3.47 (1.26)	4.39 (1.15)		
8 weeks	4.85 (1.16)	5.04 (0.90)	0.29 (0.8 to -0.21)	0.252
**Pain (0-6)**				
Baseline	3.56 (1.24)	3.63 (4.46)		
8 weeks	4.88 (1.13)	5.12 (0.98)	0.11 (0.41 to -0.64)	0.664
**Total FSFI score (2-36)**				
Baseline	19.48 (4.67)	22.28 (4.15)		
8 weeks	27.50 (5.12)	28.14 (4.77)	1.14 (3.57 to -1.28)	0.349

^a^ Mean (standard deviation)

^b^ Adjusted mean difference (95% confidence interval)

ANCOVA test adjusted for baseline score was used to compare the mean scores at post-intervention

Higher sexual function scores indicate more favorable conditions

### Sexual satisfaction

The mean (SD) sexual satisfaction was 98.35 (17.64) before the intervention and 106.44 (21.7) after the intervention in the OXT group and in the placebo group, it was 109.37 (15.94) before the intervention and 113.23 (16.48) after the intervention. After the intervention, there was no statistically significant difference between the two groups after adjusting the baseline value and age (it was borderline evidence in terms of sexual satisfaction) (AMD: 5.01; 95% CI: -0.53 to 10.56; P= 0.075). Among the five domains, only sexual contentment was significantly higher in the OXT group compared to the placebo group (AMD: 1.56; 95% CI: 0.29 to 2.83; P = 0.017). Table 3 shows the total mean sexual satisfaction and all of its domains by the groups.

**Table 3:** The mean sexual satisfaction total score and its dimensions in study groups

Variable (Score range)	Oxytocin n= 34	Placebo n= 30	AMD )95% CI)^b^	P-value
	Mean (SD)^a^	Mean (SD)^a^		
**Contentment (6-30)**				
Baseline	19.21 (4.33)	22.7 (4.6)		
8 weeks	21.56 (3.98)	23.17 (4.62)	1.56 (2.83 to 0.29)	0.017
**Communication (6-30)**				
Baseline	20.50 (3.49)	22.7 (3.39)		
8 weeks	21.23 (4.42)	23.40 (3.52)	0.23 (1.49 to -1.03)	0.716
**Coping (6-30)**				
Baseline	18.85 (5.21)	22.13 (4.31)		
8 weeks	19.68 (5.45)	22.27 (4.42)	0.12 (1.45 to -1.21 )	0.856
**Relation concern (6-30)**				
Baseline	20.26 (4.39)	21.53 (5.17)		
8 weeks	22.71 (8.65)	22.27 (4.93)	1.64 (4.67 to -1.32)	0.271
**Personal concern (6-30)**				
Baseline	19.53 (5.42)	20.23 (5.84)		
8 weeks	21.26 (5.59)	21.70 (5.11)	0.47 (1.822 to 0.9 )	0.498
**Total Sexual Satisfaction (30-150)**				
Baseline	98.35 (17.64)	109.37 (15.94)		
8 weeks	106.44 (21.7)	113.2 (16.48)	5.01 (10.56 to -0.53)	0.075

^a^ Mean (Standard Deviation)

^b^ Adjusted mean difference (95% confidence interval)

ANCOVA test adjusted for baseline score was used to compare the mean scores at post-intervention

Higher scores indicate high sexual satisfaction

### Depression

The mean (SD) depression score was 6.32 (3.8) before the intervention and 4.59 (2.40) after the intervention in the OXT group and in the placebo group, it was 7.03 (5.16) before the intervention and 6.63 (2.43) after the intervention. After the intervention, the mean depression score after adjusting the baseline value and age statistically lower in the OXT group than the placebo group (AMD: -1.90; 95% CI: -1.27 to -2.54; P < 0.001) (Table 4). The results of Mann Whitney U test showed statistically significant difference between the groups in terms of the change in depression score (EPDS at 8 weeks - EPDS at baseline) (P < 0.001).

**Table 4:** The mean depression score in study groups

Variable (Score range)	Oxytocin n= 34	Placebo n= 30	AMD (95% CI)^b^	P-value
	Mean (SD)^a^	Mean (SD)^a^		
**Depression (0-30)**				
Baseline	6.32 (3.8)	7.03 (5.16)		
8 weeks	4.59 (2.40)	6.63 (2.43)	-1.90 (-1.27 to -2.54)	<0.001

^a^ Mean (standard deviation)

^b^ Adjusted mean difference (95% confidence interval)

ANCOVA test adjusted for baseline score was used to compare the mean scores at post-intervention

The higher scores indicate the more severe condition

### Subgroup analysis based on the delivery type

In both study groups, there was no statistically significant difference between the two subgroups of women with vaginal delivery and cesarean delivery in terms of mean total scores of FSFI, sexual satisfaction and depression. Also, the interaction between study groups and delivery types was not significant (P > 0.05) (Table 5).

**Table 5:** Subgroup analysis of outcomes based on the delivery type

Variable (Score range)	Baseline		Post-intervention		Vaginal compared to cesarean delivery AMD (95% CI)^a^	Interaction between study groups and delivery types (P-value)
	Vaginal delivery (n= 27)	Cesarean delivery (n= 37)	Vaginal delivery (n= 27)	Cesarean delivery (n= 37)		
**Total FSFI score (2-36)**						0.507
Oxytocin (n= 34)	20.8 (4.3)	18.9 (4.8)	26.1 (5.9)	28.1 (4.8)	-3.1 (-6.5 to 0.4)	
Placebo (n= 30)	21.7 (4.1)	23.0 (4.3)	26.6 (4.4)	30.1 (4.7)	-2.9 (-6.2 to 0.3)	
**Total sexual satisfaction (30-150)**						0.810
Oxytocin (n= 34)	93.5 (19.7)	100.4 (16.7)	100.7 (23.2)	108.8 (21.1)	-1.1 (-10.8 to 8.5)	
Placebo (n= 30)	114.0 (13.9)	103.3 (16.9)	116.8 (14.0)	108.6 (18.9)	-2.1 (-7.8 to 3.5)	
**Depression (0-30)**						0.437
Oxytocin (n= 34)	7.0 (4.2)	7.3 (3.1)	4.3 (2.3)	4.7 (2.0)	-0.2 (-1.1 to 0.6)	
Placebo (n= 30)	6.6 (3.2)	8.5 (3.0)	6.0 (2.5)	7.4 (2.1)	-0.1 (-1.3 to 1.0)	

The data present mean (standard deviation) unless otherwise indicated

^a^ Adjusted mean difference (95% Confidence Interval)

ANCOVA test with adjusting for baseline score was used to compare the mean scores of outcomes between two types of delivery

For FSFI and sexual satisfaction, the higher score indicate the better condition and for depression, the higher scores indicate the more severe condition

### Side Effects

One participant in the OXT group and one participant in the placebo group reported mild uterine contraction and one person in the placebo group reported vaginal burning sensations. All participants in the two groups used their medication (OXT or placebo) regularly.

## Discussion

This trial found no evidence of a significant effect of OXT on the primary outcome (sexual function), but indicated some benefits on wellbeing and possibly on sexual satisfaction”.

In the present research, no evidence was found of an effect of OXT on the sexual function. We found 6 studies examining the effects of intranasal OXT on the sexual function of men and women. Even though in these studies the intranasal OXT resulted in the improvement of disparate parameters of sexual function in men and women, these changes were not statistically significant in comparison to the placebo group. Merely in some studies, it had considerable effects on the intensity of orgasm and the increase of the heart rate caused by orgasm in men [23–26, 35–37]. In the present research, placebo manifested considerable effects, which can be due to the beneficial effects of the placebo gel. The results of the present research correspond to the results of the study by Mitchell et al. (2018). They assigned 302 healthy post-menopausal women who were sexually active and had mild to severe symptoms of atrophic vaginitis into three groups; receiving vaginal estradiol, over-the-counter lubricant gel, or placebo gel. Surprisingly, the three groups manifested no statistically significant differences [38]. In accordance with the meta-analysis results of a systematic review, a considerable placebo effect has been reported in most clinical trials on female sexual function disorder. This demonstrates that 67.7% of the therapeutic effects in FSD were related to the placebo [39]. Another study by Shaughnessy et al. [40], revealed that vaginal estrogen has no superiority over lubricant placebo gel.

The results of the present research demonstrated that there was no significant difference between the two groups in terms of mean total score of sexual satisfaction and its subdomain except contentment domain. Sexual satisfaction requires normal sexual function on the basis of the stages of correspondence to the sexual stimuli (sexual desire, arousal, orgasm, and resolution) [41].

Furthermore, the results of the present research demonstrated that OXT significantly leads to the improvement of depression. The results of a study showed that intrapartum synthetic oxytocin predicted a significantly lower risk of postpartum depression [42]. Since, there is a bilateral relationship between sexual function and depression [8–10], it was expected that the effect of OXT on depression to be mediated through its effect on sexual function. As in this study, no evidence was found for the effects of OXT gel in the improvement of sexual function, it may be a direct effect (hormonal) of OXT on wellbeing. Given the results of some review studies in postpartum mothers and people with major depressive disorder reveal that there is a negative relationship between the severity of depression and the level of plasma OXT [43–45], it is suggested that OXT plasma levels to be analysed after its intravaginal administration.

During the postpartum, the level of estrogen drops due to the sudden discontinuation of placental estrogen and high levels of prolactin hormone (as a potential antagonist in the secretion of ovary estrogen) [46, 47]. This induced systematic hypoestrogenism can cause urogenital atrophy [48]. Therefore, managing atrophic vaginitis is crucial in order to preserve sexual and vaginal health and to prevent FSD. However, management of atrophic vaginitis in breastfeeding women requires a bilateral view of both mother and the infant. In addition, there are several restrictions in the systematic prescription of disparate chemical and herbal drugs for breastfeeding mothers since there is not enough information regarding the amount of secretion of the drug in mother’s milk and its effect on the milk [49–51]. Even though the standard treatment of atrophic vaginitis in breastfeeding mothers (like menopausal women) is systematic estrogen, it affects the volume of a mother’s milk, thus, the best therapeutic option for reducing or improving atrophic vaginitis in breastfeeding women are the topical products (vaginal) not the systematic products [48, 52]. Another important factor is that some medicinal vaginal creams (not just for lubricant effects) can weaken the latex in people who use barrier methods for preventing pregnancy. Therefore, the most common methods for the management of atrophic vaginitis in breastfeeding women are water-based lubricant vaginal gels [49, 53, 54].

### Limitations, strengths and implications for practice

The present research is the first study to examine the effects of OXT vaginal gel on sexual function in breastfeeding mothers while other studies focused on menopausal women. The strengths of this study include randomization, triple blinding, and no attrition resulting in low risk of bias. In the present research, standard and valid questionnaires were used for assessment of the outcomes due to the financial limitations. However, examining the laboratory criteria such as vaginal maturation could be helpful in determining the effectiveness of OXT and the subjective criteria (questionnaires) alone may not be sufficient for assessment of effectiveness of a drug. Small sample size was another limitation of this study. It is difficult to know whether the “non-significant results” are due to a lack of treatment effect, or a lack of power.

In future clinical trials, assessment of OXT effect in other women with atrophic vaginitis and sexual dysfunction such as women suffering from premature ovarian failure (POF) or women suffering from atrophic vaginitis due to abdominal or pelvic radiotherapy or chemotherapy could be considered. As no definitive conclusion can be drawn from this trial, further trials of OXT in breastfeeding women are recommended.

## Conclusions

No evidence was found for the effects of OXT gel in the improvement of sexual function, even though, OXT significantly improved sexual satisfaction in the domain of contentment, and improved the symptoms of depression in comparison to the placebo group. However, a definite conclusion requires more research in this regard.

## Supplementary Information

**Additional file 1.**

## Data Availability

The datasets used and/or analyzed during the current study available from the corresponding author on reasonable request.

## Abbreviations

OXT: Oxytocin
FSFI: Female Sexual Function Index
EPDS: Edinburgh Postpartum Depression Scale
SSSW: Sexual Satisfaction Scale for Women
AMD: Adjusted mean difference
FSD: Female sexual dysfunction
HRT: Hormone replacement therapy
HPA: hypothalamic–pituitary–adrenal
IRCT: Iranian Registry of Clinical Trials
BMI: Body mass index
POF: Premature ovarian failure
