# Supplementary material for: The effect of intra-vaginal oxytocin on sexual function in breastfeeding mothers: a randomized triple-blind placebo-controlled trial
Source: BMC Pregnancy Childbirth. 2022 Jan 22;22:62. doi: 10.1186/s12884-022-04384-w (PMC8783987; doi:10.1186/s12884-022-04384-w)
Supplement: Supplementary file 1 — Additional file 1. [file 12884_2022_4384_MOESM1_ESM.docx]

**The Edinburgh Postnatal Depression Scale**

Please select one option for each question that is the closest to how you have felt in the PAST SEVEN DAYS.

**1. I have been able to laugh and see the funny side of things:**( ) As much as I always could
( ) Not quite as much now
( ) Definitely not so much now
( ) Not at all
**2. I have looked forward with enjoyment to things:**
( ) As much as I ever did
( ) Rather less than I used to
( ) Definitely less than I used to
( ) Hardly at all
**3. I have blamed myself unnecessarily when things went wrong:**
( ) Yes, most of the time
( ) Yes, some of the time
( ) Not very often
( ) No, never
**4. I have been anxious or worried for no good reason:**
( ) No, not at all
( ) Hardly ever
( ) Yes, sometimes
( ) Yes, very often
**5. I have felt scared or panicky for no very good reason:**
( ) Yes, quite a lot
( ) Yes, sometimes
( ) No, not much
( ) No, not at all

**6. Things have been getting on top of me:**
( ) Yes, most of the time I haven’t been able to cope at all
( ) Yes, sometimes I haven’t been coping as well as usual
( ) No, most of the time I have coped quite well
( ) No, I have been coping as well as ever
**7. I have been so unhappy that I have had difficulty sleeping:**
( ) Yes, most of the time
( ) Yes, sometimes
( ) Not very often
( ) No, not at all
**8. I have felt sad or miserable:**
( ) Yes, most of the time
( ) Yes, quite often
( ) Not very often
( ) No, not at all
**9. I have been so unhappy that I have been crying:**
( ) Yes, most of the time
( ) Yes, quite often
( ) Only occasionally
( ) No, never
**10. The thought of harming myself has occurred to me:**
( ) Yes, quite often
( ) Sometimes
( ) Hardly ever
( ) Never

**Female Sexual Function Index**

**1. Over the past 4 weeks, how often did you feel sexual desire or interest?**5 = Almost always or always
4 = Most times (more than half the time)
3 = Sometimes (about half the time)
2 = A few times (less than half the time)
1 = Almost never or never
**2. Over the past 4 weeks, how would you rate your level (degree) of sexual desire or interest?**5 = Very high
4 = High
3 = Moderate
2 = Low
1 = Very low or none at all
**3. Over the past 4 weeks, how often did you feel sexually aroused ("turned on") during sexual activity or intercourse?**0 = No sexual activity

5 = Almost always or always
4 = Most times (more than half the time)
3 = Sometimes (about half the time)
2 = A few times (less than half the time)
1 = Almost never or never
**4. Over the past 4 weeks, how would you rate your level of sexual arousal ("turn on") during sexual activity or intercourse?**0 = No sexual activity
5 = Very high
4 = High
3 = Moderate
2 = Low
1 = Very low or none at all
**5. Over the past 4 weeks, how confident were you about becoming sexually aroused during sexual activity or intercourse?**0 = No sexual activity
5 = Very high confidence
4 = High confidence
3 = Moderate confidence
2 = Low confidence
1 = Very low or no confidence
**6. Over the past 4 weeks, how often have you been satisfied with your arousal (excitement) during sexual activity or intercourse?**0 = No sexual activity

5 = Almost always or always
4 = Most times (more than half the time)
3 = Sometimes (about half the time)
2 = A few times (less than half the time)
1 = Almost never or never

**7. Over the past 4 weeks, how often did you become lubricated ("wet") during sexual activity or intercourse?**0 = No sexual activity
5 = Almost always or always
4 = Most times (more than half the time)
3 = Sometimes (about half the time)
2 = A few times (less than half the time)
1 = Almost never or never
**8. Over the past 4 weeks, how difficult was it to become lubricated ("wet") during sexual activity or intercourse?**0 = No sexual activity
1 = Extremely difficult or impossible
2 = Very difficult
3 = Difficult
4 = Slightly difficult
5 = Not difficult
**9. Over the past 4 weeks, how often did you maintain your lubrication ("wetness") until completion of sexual activity or intercourse?**0 = No sexual activity
5 = Almost always or always
4 = Most times (more than half the time)
3 = Sometimes (about half the time)
2 = A few times (less than half the time)
1 = Almost never or never
**10. Over the past 4 weeks, how difficult was it to maintain your lubrication ("wetness") until completion of sexual activity or intercourse?**0 = No sexual activity
1 = Extremely difficult or impossible
2 = Very difficult
3 = Difficult
4 = Slightly difficult
5 = Not difficult
**11. Over the past 4 weeks, when you had sexual stimulation or intercourse, how often did you reach orgasm (climax)?**
0 = No sexual activity
5 = Almost always or always
4 = Most times (more than half the time)
3 = Sometimes (about half the time)
2 = A few times (less than half the time)
1 = Almost never or never
**12. Over the past 4 weeks, when you had sexual stimulation or intercourse, how difficult was it for you to reach orgasm (climax)?**0 = No sexual activity
1 = Extremely difficult or impossible
2 = Very difficult
3 = Difficult
4 = Slightly difficult
5 = Not difficult
**13. Over the past 4 weeks, how satisfied were you with your ability to reach orgasm (climax) during sexual activity or intercourse?**0 = No sexual activity
5 = Very satisfied
4 = Moderately satisfied
3 = About equally satisfied and dissatisfied
2 = Moderately dissatisfied
1 = Very dissatisfied
**14. Over the past 4 weeks, how satisfied have you been with the amount of emotional closeness during sexual activity between you and your partner?**0 = No sexual activity
5 = Very satisfied
4 = Moderately satisfied
3 = About equally satisfied and dissatisfied
2 = Moderately dissatisfied
1 = Very dissatisfied
**15. Over the past 4 weeks, how satisfied have you been with your sexual relationship with your partner?**5 = Very satisfied
4 = Moderately satisfied
3 = About equally satisfied and dissatisfied
2 = Moderately dissatisfied
1 = Very dissatisfied
**16. Over the past 4 weeks, how satisfied have you been with your overall sexual life?**5 = Very satisfied
4 = Moderately satisfied
3 = About equally satisfied and dissatisfied
2 = Moderately dissatisfied
1 = Very dissatisfied
**17. Over the past 4 weeks, how often did you experience discomfort or pain during vaginal penetration?**0 = Did not attempt intercourse
1 = Almost always or always
2 = Most times (more than half the time)
3 = Sometimes (about half the time)
4 = A few times (less than half the time)
5 = Almost never or never
**18. Over the past 4 weeks, how often did you experience discomfort or pain following vaginal penetration?**0 = Did not attempt intercourse
1 = Almost always or always
2 = Most times (more than half the time)
3 = Sometimes (about half the time)
4 = A few times (less than half the time)
5 = Almost never or never
**19. Over the past 4 weeks, how would you rate your level (degree) of discomfort or pain during or following vaginal penetration?**0 = Did not attempt intercourse
1 = Very high
2 = High
3 = Moderate
4 = Low
5 = Very low or none at all

| **Sexual satisfaction scale for women (SSS-W)** | |
| --- | --- |
| Q1: I feel content with the way my present sex life is. | 1 = Strongly disagree 2 = Disagree a little 3 = Neither agree or disagree 4 = Agree a little 5 = Strongly agree |
| Q2: I often feel something is missing from my present sex life. | 5 = Strongly disagree 4 = Disagree a little 3 = Neither agree or disagree 2 = Agree a little 1 = Strongly agree |
| Q3: I often feel I don’t have enough emotional closeness in my sex life. | 5 = Strongly disagree 4 = Disagree a little 3 = Neither agree or disagree 2 = Agree a little 1 = Strongly agree |
| Q4: I feel content with how often I presently have sexual intimacy (kissing, intercourse, etc.) in my life. | 1 = Strongly disagree 2 = Disagree a little 3 = Neither agree or disagree 4 = Agree a little 5 = Strongly agree |
| Q5: I don’t have *any* important problems or concerns about sex (arousal, orgasm, frequency, compatibility, communication, etc.). | 1 = Strongly disagree 2 = Disagree a little 3 = Neither agree or disagree 4 = Agree a little 5 = Strongly agree |
| Q6: Overall, how satisfactory or unsatisfactory is your present sex life? | 5 = Completely satisfactory 4 = Very satisfactory 3 = Reasonable satisfactory 2 = Not very satisfactory 1 = Not at all satisfactory |
| Q7: My partner often gets defensive when I try discussing sex. | 5 = Strongly disagree 4 = Disagree a little 3 = Neither agree or disagree 2 = Agree a litte 1 = Strongly agree |
| Q8: My partner and I do not discuss sex openly enough with each other, or do not discuss sex often enough. | 5 = Strongly disagree 4 = Disagree a little 3 = Neither agree or disagree 2 = Agree a little 1 = Strongly agree |
| Q9: I usually feel completely comfortable discussing sex whenever my partner wants to. | 1 = Strongly disagree 2 = Disagree a little 3 = Neither agree or disagree 4 = Agree a little 5 = Strongly agree |
| Q10: My partner usually feels completely comfortable discussing sex whenever I want to. | 1 = Strongly disagree 2 = Disagree a little 3 = Neither agree or disagree 4 = Agree a little 5 = Strongly agree |
| Q11: I have no difficulty talking about my deepest feelings and emotions when my partner wants me to. | 1 = Strongly disagree 2 = Disagree a little 3 = Neither agree or disagree 4 = Agree a little 5 = Strongly agree |
| Q12: My partner has no difficulty talking about their deepest feelings and emotions when I want him to. | 1 = Strongly disagree 2 = Disagree a little 3 = Neither agree or disagree 4 = Agree a little 5 = Strongly agree |
| Q13: I often feel my partner isn’t sensitive or aware enough about my sexual likes and desires. | 5 = Strongly disagree 4 = Disagree a little 3 = Neither agree or disagree 2 = Agree a little 1 = Strongly agree |
| Q14: I often feel that my partner and I are not sexually compatible enough. | 5 = Strongly disagree 4 = Disagree a little 3 = Neither agree or disagree 2 = Agree a little 1 = Strongly agree |
| Q15: I often feel that my partner’s beliefs and attitudes about sex are too different from mine. | 5 = Strongly disagree 4 = Disagree a little 3 = Neither agree or disagree 2 = Agree a little 1 = Strongly agree |
| Q16: I sometimes think my partner and I are mismatched in needs and desires concerning sexual intimacy. | 5 = Strongly disagree 4 = Disagree a little 3 = Neither agree or disagree 2 = Agree a little 1 = Strongly agree |
| Q17: I sometimes feel that my partner and I might not be physically attracted to each other enough. | 5 = Strongly disagree 4 = Disagree a little 3 = Neither agree or disagree 2 = Agree a little 1 = Strongly agree |
| Q18: I sometimes think my partner and I are mismatched in our sexual styles and preferences. | 5 = Strongly disagree 4 = Disagree a little 3 = Neither agree or disagree 2 = Agree a little 1 = Strongly agree |
| Q19: I’m worried that my partner will become frustrated with my sexual difficulties. | 5 = Strongly disagree 4 = Disagree a little 3 = Neither agree or disagree 2 = Agree a little 1 = Strongly agree |
| Q20: I’m worried that my sexual difficulties will adversely affect my relationship. | 5 = Strongly disagree 4 = Disagree a little 3 = Neither agree or disagree 2 = Agree a little 1 = Strongly agree |
| Q21: I’m worried that my partner may have an affair because of my sexual difficulties. | 5 = Strongly disagree 4 = Disagree a little 3 = Neither agree or disagree 2 = Agree a little 1 = Strongly agree |
| Q22: I’m worried that my partner is sexually unfulfilled. | 5 = Strongly disagree 4 = Disagree a little 3 = Neither agree or disagree 2 = Agree a little 1 = Strongly agree |
| Q23: I’m worried that my partner views me as less of a woman because of my sexual difficulties. | 5 = Strongly disagree 4 = Disagree a little 3 = Neither agree or disagree 2 = Agree a little 1 = Strongly agree |
| Q24: I feel like I’ve disappointed my partner by having sexual difficulties. | 5 = Strongly disagree 4 = Disagree a little 3 = Neither agree or disagree 2 = Agree a little 1 = Strongly agree |
| Q25: My sexual difficulties are frustrating to me. | 5 = Strongly disagree 4 = Disagree a little 3 = Neither agree or disagree 2 = Agree a little 1 = Strongly agree |
| Q26: My sexual difficulties make me feel sexually unfulfilled. | 5 = Strongly disagree 4 = Disagree a little 3 = Neither agree or disagree 2 = Agree a little 1 = Strongly agree |
| Q27: I’m worried that my sexual difficulties might cause me to seek sexual fulfillment outside my relationship. | 5 = Strongly disagree 4 = Disagree a little 3 = Neither agree or disagree 2 = Agree a little 1 = Strongly agree |
| Q28: I’m so distressed about my sexual difficulties that it affects the way I feel about myself. | 5 = Strongly disagree 4 = Disagree a little 3 = Neither agree or disagree 2 = Agree a little 1 = Strongly agree |
| Q29: I’m so distressed about my sexual difficulties that it affects my own well-being. | 5 = Strongly disagree 4 = Disagree a little 3 = Neither agree or disagree 2 = Agree a little 1 = Strongly agree |
| Q30: My sexual difficulties annoy and anger me. | 5 = Strongly disagree 4 = Disagree a little 3 = Neither agree or disagree 2 = Agree a little 1 = Strongly agree |
